# Measurements of the anatomical structures of the mandibular buccal shelf for the safe insertion of mini-implants: a cone-beam computed tomography retrospective study

**DOI:** 10.1186/s12903-025-06980-6

**Published:** 2025-10-14

**Authors:** Xing Fan, Lixian Yuan, Zhiwei Wang, Ke Liu, Baiting Luan, Yi Wen, Xin Liu

**Affiliations:** 1 Department of Orthodontics, Yanan University Affiliated Hospital, Yanan, 716000 China; 2 Department of Preventive Dentistry, School of Stomatology, The Fourth Military Medical University, State Key Laboratory of Oral & Maxillofacial Reconstruction and Regeneration, National Clinical Research Center for Oral Diseases, Shaanxi Clinical Research Center for Oral Diseases, Xi‘an, 710032 China; 3 Department of Orthodontics, School of Stomatology, The Fourth Military Medical University, State Key Laboratory of Oral & Maxillofacial Reconstruction and Regeneration, National Clinical Research Center for Oral Diseases, Shaanxi Clinical Research Center for Oral Diseases, No. 145, Changle West Road, Xincheng District, Xi‘an, Shanxi 710032 China

**Keywords:** Mandibular buccal shelf, Mini-implant, Insertion length, Insertion angle

## Abstract

**Background:**

The purpose of this study was to obtain measurements of the anatomical structures of the mandibular buccal shelf (MBS) using cone-beam computed tomography (CBCT) for the safe insertion of orthodontic mini-implants.

**Methods:**

The sample consisted of the CBCT records of 100 subjects. The scans were imported into a reconstruction program. Measurements were taken in the coronal plane for three different roots: the mandibular first molar distal root (6D), second molar mesial root (7 M), and second molar distal root (7D). The inferior alveolar nerve canal (IANC) was used as a reference to determine the safe insertion depth. The roots of the mandibular molars were used as a reference to determine the maximum safe insertion angle. Analysis of variance with post hoc analysis and Kruskal-Wallis H test with post hoc analysis were used for data analysis.

**Results:**

For the 6Ds, the average bone depth was 20.33 ± 3.6 mm, the average depth above the IANC plane was 9.55 ± 2.97 mm, and the average maximum safe insertion angle was 47.47 ± 18.94°. For the 7Ms, the average bone depth was 22.06 ± 2.85 mm, the average depth above the IANC plane was 10.71 ± 2.67 mm, and the average maximum safe insertion angle was 43.14 ± 15.26°. For the 7Ds, the average bone depth was 23.75 ± 2.86 mm, the average depth above the IANC plane was 12.12 ± 2.61 mm, and the average maximum safe insertion angle was 28.39 ± 10.96°. From anterior to posterior, the buccal bone depth and depth above the IANC gradually increased (*P* < 0.01), there was no significant difference in the maximum safe insertion angle between the 6Ds and 7Ms (*P* = 1.000), but the significant difference was observed between 6Ds and 7Ds, 7Ms and 7Ds (*P* < 0.000).

**Conclusions:**

The optimal implantation site for mini-implant in the MBS is at the buccal site corresponding to the second molar. A maximum insertion length of ≤ 12 mm and a maximum insertion angle which respects the long axial of the tooth root of ≤ 28° are recommended for the safe insertion of mini-implants in the MBS.

## Introduction

Mini-implants are used in clinical orthodontic treatment because of their small size, ease of insertion and removal, minimal degree of associated surgical trauma, and the ability to apply directional orthodontic force and provide absolute orthodontic anchorage [1, 2]. However, following their placement, loosening and failure may occur, and reimplantation requires that the patient undergo repeat invasive procedures, ‌with no guarantee of preventing recurrent loosening or failure [3]. These issues remain the most troubling challenges for clinicians. Current data show an overall failure rate of 13.5% for mini-implants [4, 5], with those placed in the palatal region showing an immediate failure rate of 5% [6], while those placed in the mandibular buccal shelf (MBS) have a failure rate of approximately 7% [7]. The rate of successful mini-implant placement is closely related to the anatomical characteristics of the insertion site [8].

The MBS is located on the buccal side of the posterior mandibular body. Compared with the root of the mandibular molars, the MBS has sufficient bone volume for implantation [7]. Moreover, placement of implants in the MBS avoids interference with orthodontic tooth movements (e.g., mandibular dentition distalization) while ‌demonstrating reduced risks of injury to the roots [9–11]. Therefore, it is an optimal site for the placement of mini-implants. However, there are substantial anatomical differences in bone size and the site of the inferior alveolar nerve canal (IANC) at different locations along the MBS [12]. Changes in the width and inclination of the bone platform in the MBS can significantly influence the optimal site and angle of placement of mini-implants. Moreover, the different insertion sites and angles may impart different risks of damage to the corresponding alveolar nerve and root [13–15]. To identify a safe approach for the mini-plant placement, the computer-guided systems-including static computer-guided technique and dynamic computer-guided surgery-have been used to achieve accurate implant placement while minimizing failure rates and complications [6, 16–18]. However, individual anatomical variability remains a crucial factor that must be considered during mini-implant placement [18].

To date, many scholars have studied the anatomy of the MBS through CBCT images. The use of CBCT has been available for over a decade. CBCT can provide three-dimensional (3D) imaging with minimum distortion, but the amount of radiation exposure is high compared to two-dimensional (2D) radiography [19]. Nevertheless, intricate anatomical structures demand the use of CBCT because it is the most reliable modality in such cases [20]. Therefore, investigating anatomical structure in the MBS can provide orthodontists with some useful information. However, current research has focused mainly on bone depth, and thickness [21, 22], without providing clinical guidelines on safe insertion lengths and angles for mini-implants. The placement of mini-implants in the MBS is usually based on clinical experience [23]. The aim of this study was to determine the optimal mini-implant insertion position, that is, the position that offers a safe insertion depth and angle, at three different sites on the buccal side 4 mm from the CEJ to provide a reference for the clinical application of mini-implants in MBS.

## Methods

The clinical investigation was in accordance with the ethical principles of the Declarations of Helsinki. The patients or their guardians were informed of the content, risks, and benefits of the study, and written consent was obtained. This retrospective study was approved by the Research Ethics Committee of The Fourth Military Medical University. The work followed the guidelines of the Reporting of Observational Studies in Epidemiology (STROBE) for observational studies [24]. The sample size was calculated using the formula described by Pandis [25], based on a study power of 95% (α = 0.05). The inclusion criteria included: (1) skeletal maturity of craniofacial was completed, as assessed by cervical vertebral maturation method [26]; (2) fully erupted mandibular second molars; (3) need of orthodontic treatment, with no CBCT images obtained solely for research purposes; (4) the correct visualization of the buccal shelf. The exclusion criteria were as follows: (1) previous orthodontic treatment; (2) extraction of the mandibular first or second molars; (3) implants or pontics replacing the mandibular first or second molars; (4) periodontal disease, history of orthognathic surgery, or any genetic syndrome; (5) evident asymmetries; and (6) CBCT showing supernumerary teeth, impacted teeth, enlarged cystic follicles, or any other pathology in the area of interest. These exclusion criteria were derived from the article published by Eto [27]. The sample consisted of the CBCT records of 100 subjects. The average age of the subjects was 26.2 ± 5.5 years.

**Fig. 1:**
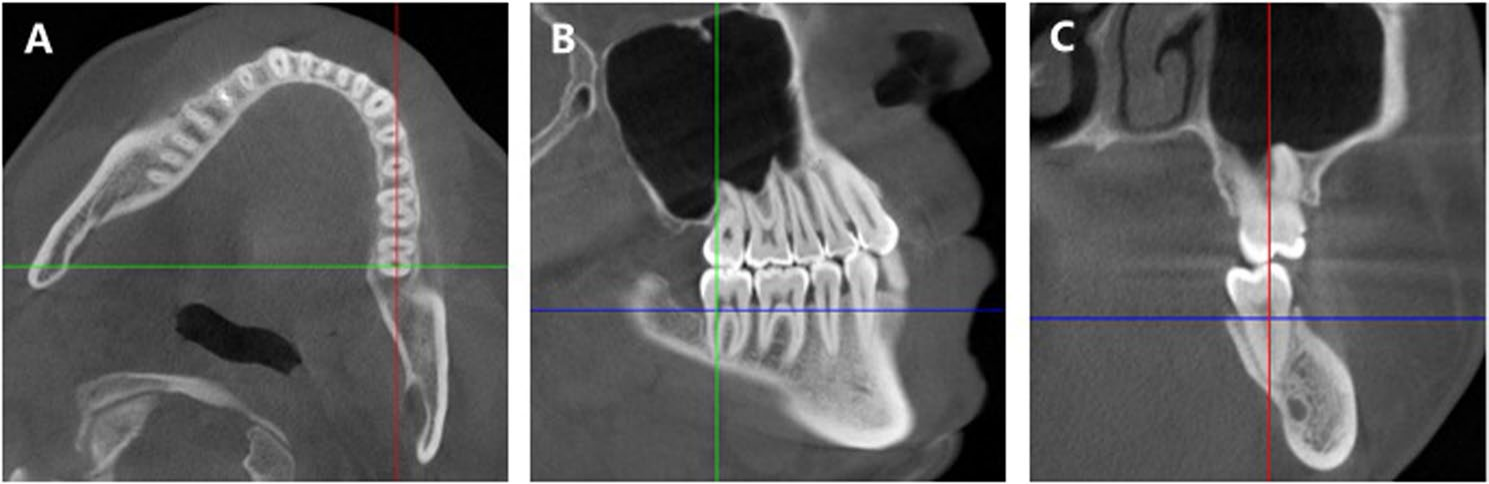
Software interface (coronal: green line; sagittal: red line; axial: blue line). **A** The red line passes through the root furcations of the left first and second molars. **B** The green line passes through the long axis of the root to be evaluated. **C** The coronal plane was determined on the basis of the axial and sagittal planes

First, CBCT images were obtained. The acquisition parameters of CBCT (LARGEV, Beijing, China) was set as follows: 100 kV, 6 mA, 16 × 15 cm field of view with 0.25 mm slice thickness and 26 s acquisition time. All the images were analyzed with Dolphin Imaging software 11.8 (Dolphin Imaging and Management Solutions, Chatsworth, Calif). To minimize measurement errors caused by differences in head posture, all the images were oriented using the following procedure. Initially, the axial plane was oriented so that the red line drawn in Dolphin Imaging software passed through the root furcations of the mandibular left molars (Fig. 1, A). Then, the sagittal plane was oriented so that the green line passed through the long axis of the molar root to be evaluated (Fig. 1, B). Finally, the coronal plane was determined on the basis of the axial and sagittal planes (Fig. 1, C). Measurements were taken in the coronal plane for the distal root of the mandibular left first molar (6D), the mesial root of the mandibular left second molar (7 M) and the distal root of the mandibular left second molar (7D).

**Fig. 2:**
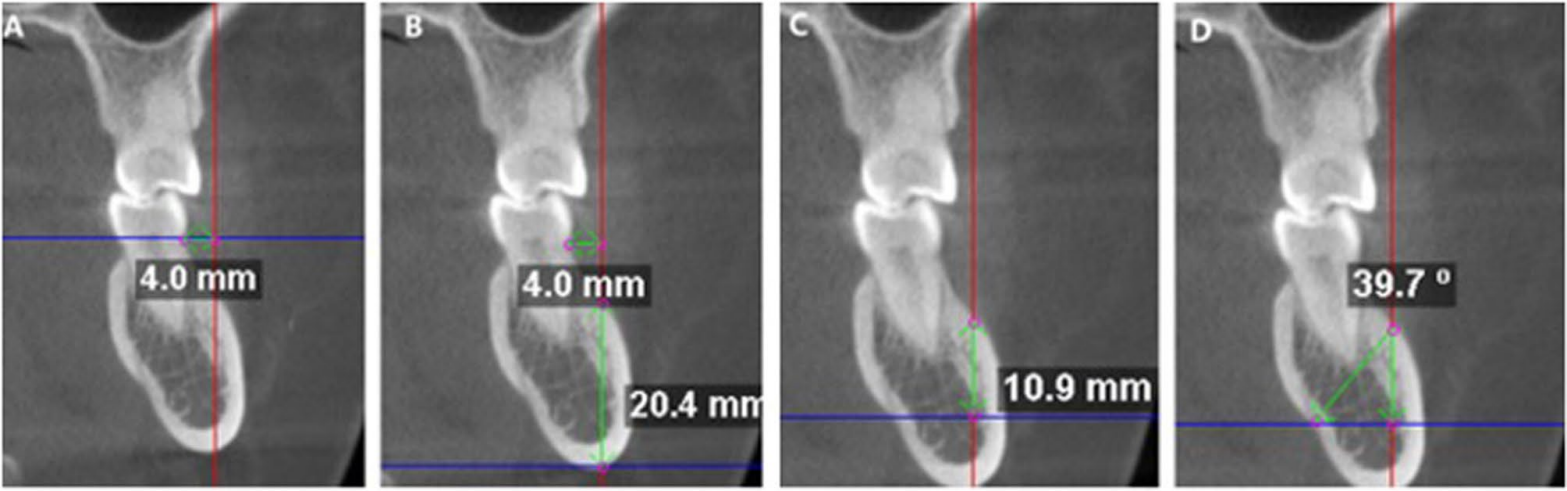
Measurement of the safe insertion depth and insertion angle for the MBS. **A** 4 mm buccal to the CEJ; **B** Depth of the MBS; **C** Perpendicular distance from the insertion site to the IANC plane; **D** Maximum safe insertion angle from the insertion site to the root apex of the tooth

The depth of the buccal bone was evaluated 4 mm from the CEJ on the buccal side (Fig. 2, A) [21, 22]. First, we measured the bone depth of the MBS (Fig. 2, B). We used the IANC as a reference for determining the safe insertion depth for the MBS. A horizontal reference plane was drawn from the roof of the IANC. Then, a perpendicular line was drawn from the insertion point to the plane of the IANC; this distance represents the safe insertion depth in the MBS (Fig. 2, C). The safe insertion angle was measured when the direction of the insertion angle is tangent to the root apex of the teeth and the maximum safe insertion angle for the MBS was marked (Fig. 2D). The procedure was repeated for all three roots in the coronal plane for each scanned image. Owing to the symmetry of both the left and right MBSs in our study, measurements were made only on the left side.

### Statistical analysis

All the statistical analyses were conducted using SPSS 25.0 (IBM, USA), and the significance level for all the tests was set to *P* < 0.05. Descriptive statistics were performed, including the mean, standard deviation (SD), and maximum and minimum values of all measurement data from the root scan sections (Table 1). The descriptive statistics were used to preliminarily evaluate potentially suitable insertion sites, safe insertion depths and safe insertion angles.

**Table 1:** Descriptive statistics of the MBS bone depth, safe insertion depth and maximum safe insertion angle

		*N*	Mean	SD	Min	Max
Bone depth (mm)	6D	67	20.33	3.36	13.10	29.10
	7 M	94	22.10	2.88	14.80	29.60
	7D	87	23.75	2.86	18.50	30.80
Distance to IANC (mm)	6D	67	9.55	2.97	3.60	18.50
	7 M	97	10.71	2.67	3.80	18.00
	7D	100	12.12	2.61	5.60	20.80
Insertion Angle (°)	6D	67	47.47	18.94	12.50	89.10
	7 M	97	43.14	15.26	15.70	86.40
	7D	100	28.39	10.96	6.40	78.20

Then, Variance homogeneity analysis was performed on all data. It showed that the data in the insertion angle group was found not conform to a normal distribution (Table 2), while the data from the bone depth and distance to IANC groups were found conform to a normal distribution. ANOVA and Post hoc analysis were used for the data from the bone depth and distance to IANC groups (Tables 3 and 4). The result showed that there was significant difference in the bone depth and distance to IANC among the 6Ds, 7Ms and 7Ds (*P* < 0.01). Kruskal-Wallis H test with post hoc analysis was used for the data from the insertion angle group (Tables 5 and 6). The result showed that there was no significant difference in the maximum safe insertion angle between the 6Ds and 7Ms (*P* = 1.000), while significant difference was observed between 6Ds and 7Ds, 7Ms and 7Ds (*P* < 0.000). Interobserver reliability was assessed by measuring the insertion depths and angles for the MBS in 20 randomly selected subjects again after 14 days. A paired student t test was used to assess interobserver reliability. Intraclass correlation showed good reliability (*r* = 0.95).

**Table 2:** The results of variance homogeneity analysis in different groups

	6D	7 M	7D	*P* value
Insertion depth (mm)	20.33 ± 3.6	22.06 ± 2.85	23.75 ± 2.86	0.359
Distance to IANC (mm)	9.55 ± 2.97	10.71 ± 2.67	12.12 ± 2.61	0.626
Insertion angle (°)	47.47 ± 18.94°	43.14 ± 15.26°	28.39 ± 10.96°	0.000

**Table 3:** ANOVA for bone depth and distance to IANC

	6D	7 M	7D	*P* value
Insertion depth (mm)	20.33 ± 3.6	22.06 ± 2.85	23.75 ± 2.86	0.000
Distance to IANC (mm)	9.55 ± 2.97	10.71 ± 2.67	12.12 ± 2.61	0.000

**Table 4:** Post hoc analysis for bone depth and distance to IANC

Insertion depth (mm)	Location	*P* value
6D	7 M	0.000
	7D	0.000
7 M	6D	0.000
	7D	0.000
7D	6D	0.000
	7 M	0.000
Distance to IANC (mm)		
6D	7 M	0.008
	7D	0.000
7 M	6D	0.008
	7D	0.000
7D	6D	0.000
	7 M	0.000

**Table 5:** Kruskal-Wallis H test for insertion angle

	6D	7 M	7D	*P* value
Insertion angle (°)	47.47 ± 18.94°	43.14 ± 15.26°	28.39 ± 10.96°	0.000

**Table 6:** Post hoc analysis for insertion angle

Insertion angle (°)	*P* value
7D-7 M	0.000
7D-6D	0.000
7 M-6D	1.000

## Results

**Fig. 3:**
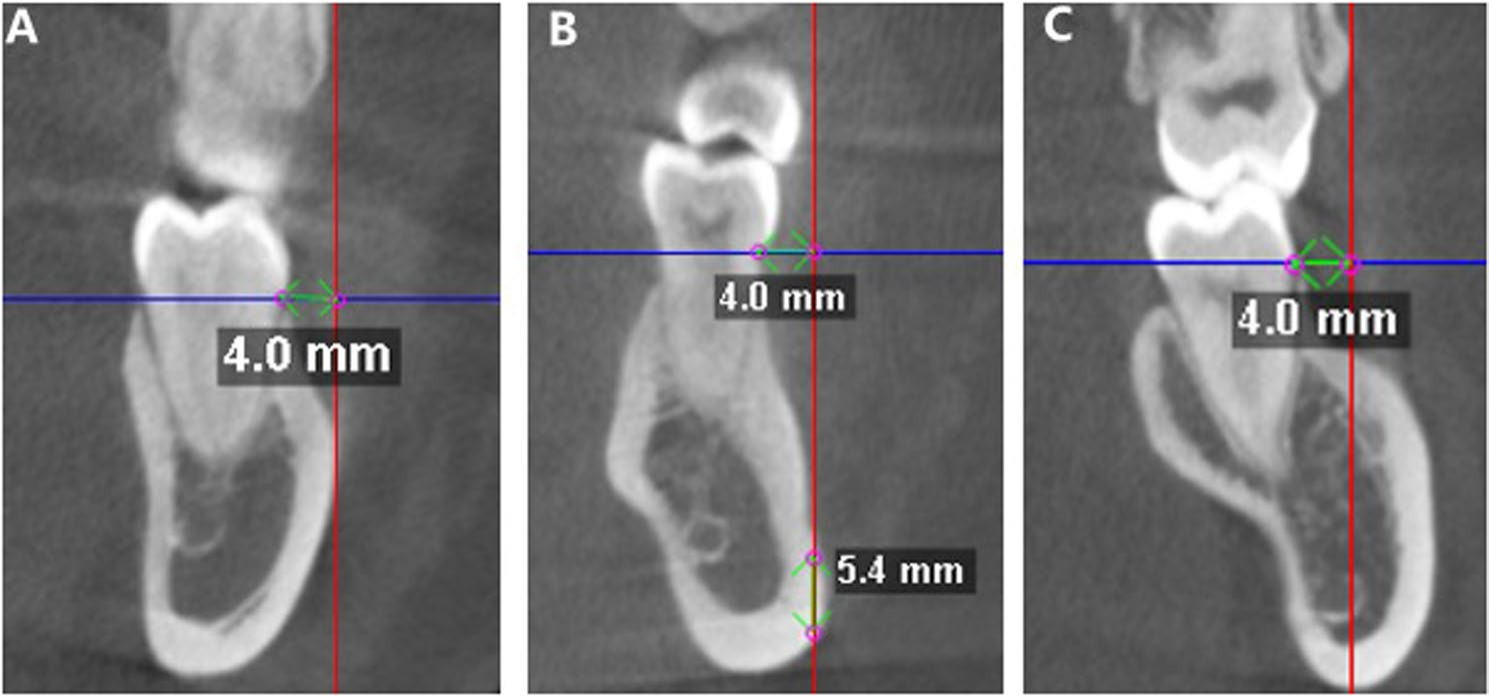
Special conditions in the measurement of the MBS. **A** The insertion point 4 mm buccal to the CEJ is outside the MBS; **B** The insertion point 4 mm buccal to the CEJ is below the root apex level and the IANC plane; **C** Choosing an insertion point 4 mm buccal to the CEJ may result in penetration of the IANC

For the 6Ds, the insertion point 4 mm buccally from the CEJ was outside the MBS in 15% of cases (Fig. 3, A) and below the root apex level in 18% of cases, among which 2% were below the IANC (Fig. 3, B). Thus, a total of 33% of the insertion sites were unavailable. For the remaining cases, the average bone depth was 20.33 ± 3.6 mm, the average depth above the IANC plane was 9.55 ± 2.97 mm, and the average maximum safe insertion angle was 47.47 ± 18.94°. For the 7Ms, the insertion point located 4 mm buccally from the CEJ was outside the MBS in 1% of cases and below the root apex level in 2% of cases. Thus, a total of 3% of all the insertion sites were unavailable. For the remaining cases, the average bone depth was 22.06 ± 2.85 mm, the average depth above the IANC plane was 10.71 ± 2.67 mm, and the average maximum safe insertion angle was 43.14 ± 15.26°. The insertion point 4 mm buccally from the CEJ was available for all 7Ds, for which the average bone depth was 23.75 ± 2.86 mm. In 13% of cases, the insertion would penetrate the IANC (Fig. 3, C). The average depth above the IANC plane was 12.12 ± 2.61 mm, and the average maximum safe insertion angle was 28.39 ± 10.96°.

## Discussion

Recent years, many studies have focus on the safe insertion of mini-implants and the optimal insertion site in the mandibular buccal shelf [14, 28]. However, the research on the safe insertion lengths and angles for mini-implants, on the inferior alveolar nerve which is a key anatomical structure in the MBS, are limited [12]. The purpose of this study is to investigate the safe insertion length and angle for mini-implants through anatomical measurements to provide a theoretical reference for clinical application. Previous studies considered 4 mm buccal bone thickness to be the minimum value for safe mini-implants insertion in the MBS [29]. 4 mm buccal to the CEJ at the distal root of the second molar was the optimal insertion site in the MBS [21, 22]. So, in this study, 4 mm from the CEJ was chosen across all sites. Previous studies states that cortical bone thickness between the left and right sides of an individual is similar in the MBS [12, 30]. In the study, only patients demonstrating a symmetrical MBS were selected, so the measurements were obtained only on the left side [31, 32].

The research data of this study revealed that at 6D, the bone volume 4 mm from the buccal side of the CEJ was unavailable in 33% of cases, while this was the case only for 3% of the 7Ms. In contrast, insertion sites 4 mm from the buccal side of the CEJ were available for all examined 7Ds. These results indicate that the bone volume at the buccal side of the MBS gradually increased in the posterior direction, which is similar to the findings of many previous studies [22, 27]. Another observation result is that at a distance 4 mm from the buccal side of the CEJ, when the insertion path was parallel to the long axial of the tooth root, the IANC was not penetrated for both 6D and 7 M, while at 7D, the insertion path penetrated the IANC in 13% of cases. These results indicate that as the insertion site moves distally, the insertion path gets closer to the IANC, greatly increasing the risk of damage to this structure.

In addition, at 6D, the average bone depth was 20.33 ± 3.6 mm, and the average depth above the IANC plane was 9.55 ± 2.97 mm. At 7 M, the average bone depth was 22.06 ± 2.85 mm, and the average depth above the IANC plane was 10.71 ± 2.67 mm. At 7D, the average bone depth was 23.75 ± 2.86 mm, and the average depth above the IANC plane was 12.12 ± 2.61 mm. These results indicate that from 6D to 7D, the buccal bone depth and depth above the IANC gradually increase (*P* < 0.000). Then the tooth root corresponding to the insertion site was used as a reference for measuring the safe insertion angle. When the mini-implants are inserted at a certain angle to the long axis of the teeth root, there is a risk of root contact. The minimum angle at which root contact occurs was calculated and defined it as the maximum safe insertion angle. The insertion angle respects the long axial of the tooth root. The average maximum safe insertion angles at 6D, 7 M, and 7D were 47.47 ± 18.94°, 43.14 ± 15.26°, and 28.39 ± 10.96°, respectively. These results indicate that in the MBS, the insertion angle needs to be smaller when moving distally. Although the bone volume becomes more sufficient in the distal region, it consequently only allows smaller insertion angles, even angles parallel to the long axis of the tooth root. Conversely, when moving mesially, the bone volume becomes insufficient, and the mini-implants may require a larger angle for insertion. This finding aligns with clinical realities. In oral surgery, implantation at posterior sites is technically challenging due to limited visibility and accessibility, and smaller insertion angles may simplify implantation in these areas. While anterior regions provide better visibility and easier placement, the lower bone volume in these regions requires a larger insertion angle. However, as the insertion site moves distally, there may be a greater risk of damage to the IANC, so the insertion length must be carefully controlled to avoid nerve damage.

This study focused on evaluating the safe insertion length and angle of mini-implants in the MBS. It also has some limitations. In this study, only the left side of the MBS was evaluated. However, bone density and the course of the IANC may not bilateral symmetry sometimes and the significant differences in skeletal characteristics are observed among individuals. Further study should be taken in the future. The results of this study should only be used as a general reference and not as a final set of guidelines. In clinical practice, CBCT should be used to determine the ideal insertion site, length, and angle for individual cases if the conditions permit.

## Conclusions

1. Both the buccal bone depth and the depth above the IANC gradually increase in the posterior direction in the MBS.
2. The maximum safe insertion angle gradually decreases in the posterior direction in the MBS.
3. The optimal site for mini-implants insertion in the MBS is at the buccal site corresponding to the second molar.
4. For placing mini-implants safely in the MBS, we recommend a maximum insertion length ≤ 12 mm and a maximum insertion angle ≤ 28°.

## Data Availability

All data generated or analysed during this study are included in this published article.
